# Galectin-3 Binds to Lubricin and Reinforces the Lubricating Boundary Layer of Articular Cartilage

**DOI:** 10.1038/srep25463

**Published:** 2016-05-09

**Authors:** Heidi L. Reesink, Edward D. Bonnevie, Sherry Liu, Carolyn R. Shurer, Michael J. Hollander, Lawrence J. Bonassar, Alan J. Nixon

**Affiliations:** 1 Department of Clinical Sciences, College of Veterinary Medicine, Cornell University, Ithaca, NY, United States of America; 2 Sibley School of Mechanical and Aerospace Engineering, Cornell University, Ithaca, NY, United States of America; 3 School of Chemical and Biomolecular Engineering, Cornell University, Ithaca, NY, United States of America; 4 Meinig School of Biomedical Engineering, Cornell University, Ithaca, NY, United States of America

## Abstract

Lubricin is a mucinous, synovial fluid glycoprotein that enables near frictionless joint motion via adsorption to the surface of articular cartilage and its lubricating properties in solution. Extensive *O-*linked glycosylation within lubricin’s mucin-rich domain is critical for its boundary lubricating function; however, it is unknown exactly how glycosylation facilitates cartilage lubrication. Here, we find that the lubricin glycome is enriched with terminal β-galactosides, known binding partners for a family of multivalent lectins called galectins. Of the galectin family members present in synovial fluid, we find that galectin-3 is a specific, high-affinity binding partner for lubricin. Considering the known ability of galectin-3 to crosslink glycoproteins, we hypothesized that galectins could augment lubrication via biomechanical stabilization of the lubricin boundary layer. We find that competitive inhibition of galectin binding results in lubricin loss from the cartilage surface, and addition of multimeric galectin-3 enhances cartilage lubrication. We also find that galectin-3 has low affinity for the surface layer of osteoarthritic cartilage and has reduced affinity for sialylated *O*-glycans, a glycophenotype associated with inflammatory conditions. Together, our results suggest that galectin-3 reinforces the lubricin boundary layer; which, in turn, enhances cartilage lubrication and may delay the onset and progression of arthritis.

Lubricin is a large, mucinous glycoprotein critical for boundary lubrication of articular cartilage. Lubricin protects against cartilage damage, with intra-articular lubricin supplementation mitigating the severity and progression of post-traumatic arthritis in animal models^1^,^2^,^3^,^4^,^5^, and patients with a genetic deficiency in lubricin production developing precocious multiple joint failure^6^. Originally isolated from synovial fluid^7^ and expressed by synovial fibroblasts^8^ or as the variant superficial zone protein by superficial zone chondrocytes^8^, lubricin is a semi-flexible rod with an extensive mucin-rich central domain. The mucin-rich domain of lubricin is composed of a repeating degenerate sequence of EPAPTTK residues that undergo extensive glycosylation (30–50% w/w basis) with *O-*linked oligosaccharide chains^9^,^10^. Effective boundary lubrication by lubricin is dependent upon both its ability to adsorb to articular cartilage^11^,^12^ and the presence of *O-*linked β(1–3)Gal-GalNAc oligosaccharides^13^; however, it is still not fully understood how lubricin interacts with other cartilage matrix or synovial fluid components and how the boundary layer is either stabilized or replenished during the lubrication cycle.

Glycans are in large part responsible for the boundary lubricating properties of lubricin. Mucinous glycoproteins, like lubricin, are defined by their densely clustered pendant glycans that initiate with N-acetylgalactosamine saccharides covalently linked to the polypeptide through the oxygen molecules of serine and threonine side chains (*O*-linked). Site-specific glycan analysis of lubricin identified 168 independent *O-*glycosylation sites^10^, of which galactosamine, galactose and N-acetylneuraminic acid comprise 98% of the carbohydrate residues^9^. Although the specific mechanisms whereby lubricin mediates boundary lubrication are still debated, several authors propose that repulsive hydration forces^14^,^15^ or charge repulsion^15^, mediated by the negatively charged sialic acids of lubricin’s glycans, are primary forces governing lubrication. However, removal of sialic acids alone only decreased boundary lubrication by 19.3%^13^ whereas partial removal of β(1–3)Gal-GalNAc oligosaccharides resulted in a 77.2% reduction in lubricating ability^13^. These findings suggest that glycans may have functions in lubrication beyond their accepted roles in hydration and charge repulsion.

Changes in glycosylation are a hallmark of disease states characterized by chronic inflammation^16^. Site-specific glycopeptide analysis of lubricin has revealed heterogeneity in glycan structure at specific sites within lubricin’s mucin-rich domain^10^, and significant variations in the concentration of sialic acid residues on lubricin have been measured in clinical samples^17^. Recent studies characterizing synovial fluid lubricin from patients with osteoarthritis (OA) and rheumatoid arthritis (RA)^18^ and synovial fluid lubricin from horses with normal joints, OA joints and osteochondral fragmentation^19^ have revealed changes in the glycosylation of lubricin in disease. Given the importance of lubricin’s *O-*linked β(1–3)Gal-GalNAc oligosaccharides^20^ in mediating cartilage lubrication, inflammation-induced changes in the glycosylation of lubricin could potentially have a major impact on its interaction with other synovial fluid constituents and boundary lubricating ability^10^,^18^.

A considerable body of evidence is accumulating to suggest that lubricin may be an effective therapy for the treatment of osteoarthritis (OA). Lubricin synovial fluid concentrations are decreased in experimental rodent models of post-traumatic OA, including the anterior cruciate ligament (ACL)-deficient guinea pig stifle^21^ and the ACL-deficient rat stifle^22^. Furthermore, replenishing lubricin, either through gene therapy^2^ or administration of recombinant lubricin^3^,^4^,^5^ delays the development and progression of arthritis in rodent OA models. However, despite the critical role that *O-*linked glycosylation plays in mediating boundary lubrication, little effort has been devoted to characterizing the glycosylation of administered recombinant lubricin or the glycoprotein product of lubricin gene therapy approaches. Thus, it is unknown what glycan composition of lubricin could be used clinically to derive the optimal therapeutic benefit.

In biology, a major function of glycans is to mediate biomolecular interactions; however, little attention has been focused on elucidating mechanisms by which glycans may facilitate lubrication distinct from repulsive or hydration mechanisms. Glycans mediate protein-protein interactions through lectins such as siglecs, selectins and galectins. Lectin glycan interactions play a critical role in several biological contexts, ranging from lattice formation to receptor signalling to glycoprotein clustering and assembly^23^,^24^,^25^.

Therefore, our objective was to examine the lubricin glycome in order to identify potential synovial fluid binding partners for lubricin. Based upon the enrichment of terminal β-galactoside residues and the high density of β(1–3)Gal-GalNAc T-antigens in normal equine synovial fluid lubricin, combined with the abundant expression of galectins-1 and -3 in cartilage and synovial fluid^26^,^27^, we hypothesized that galectins may be binding partners for lubricin. Here, we find that galectin-3 is a specific, high-affinity binding partner for lubricin, capable of enhancing boundary lubrication by cross-linking oligosaccharide moieties on lubricin and stabilizing the lubricin lattice.

## Results

### Glycophenotype of the Articular Cartilage Boundary Layer

**Figure 1:**
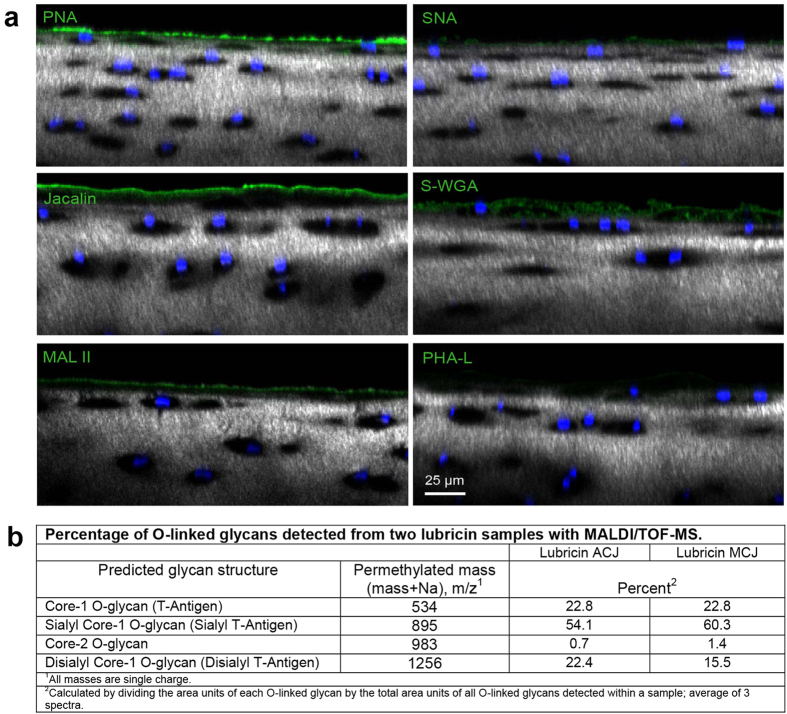
The glycophenotype of lubricin. (**a**) Healthy equine articular cartilage imaged with confocal and multiphoton microscopy (40X) following incubation with fluorophore-conjugated lectins. Chondrocyte nuclei are stained with Hoechst 33342 (blue), and collagen is imaged using second harmonic generation microscopy (grey). PNA boundary layer staining indicates the presence of nonsialylated core-1 O-glycans (Galβ(1–3)GalNAc). Both MAL II, which preferentially binds to α2–3 sialylated core-1 *O-*glycans, and jacalin, which binds to both sialylated and nonsialylated core-1 *O-*glycans, labelled the boundary layer of articular cartilage. Faint staining of the superficial zone interterritorial matrix with S-WGA was present, whereas no appreciable boundary staining was present for either S-WGA or PHA-L, demonstrating that core-2 *O-*glycans and complex, branched *N-*glycans do not contribute substantially to the boundary layer oligosaccharide layer. Lectins: PNA, peanut agglutinin; jacalin; MAL II, *Maackia amurensis* lectin II; SNA, *Sambucus nigra;* S-WGA, S-wheat germ agglutinin; PHA-L,leucoagglutinin. (**b**) Relative ion intensity of O-linked oligosaccharides detected by MALDI/TOF-mass spectrometry in two healthy equine synovial fluid samples. Monosialylated structures predominate, followed by nearly equal distributions of disialylated and nonsialylated core-1 O-glycans.

In order to determine what glycans were present on healthy cartilage, adult equine articular cartilage explants were incubated with fluorophore-conjugated lectins followed by nuclear staining with Hoechst 33342 and combined confocal and multiphoton microscopy (Fig. 1A). Peanut agglutinin (PNA), which binds to nonsialylated core-1 *O-*glycans, intensely stained the cartilage boundary layer. *Maackia amurensis* lectin II (MALII), which preferentially binds to α2–3 sialylated core-1 *O-*glycans, and jacalin, which binds to both sialylated and nonsialylated core-1 *O-*glycans, also labelled the surface layer of articular cartilage. Although there was faint staining of the superficial zone interterritorial matrix with succinylated wheat germ agglutinin (S-WGA) and phytohemagluttinin-leucoagglutinin (PHA-L), no appreciable boundary staining was present for either of these lectins, indicating that core-2 *O-*glycans and complex, branched *N-*glycans do not contribute substantially to the oligosaccharide population of the cartilage boundary layer. Weak staining of the cartilage surface by *Sambucus nigra* (SNA) was detected, indicating the presence of low quantities of α2–6 linked sialic acids. Taken together, these results suggest that the articular cartilage boundary layer is composed of a significant proportion of nonsialylated core-1 *O-*glycans.

Lubricin is the predominant carrier of O-linked glycans within the cartilage boundary layer. The glycosylation profiles of lubricin from human patients with OA and RA have been described^10^,^18^,^28^. To further characterize lubricin’s O-linked glycans, equine synovial fluid lubricin obtained from the healthy carpal joints of a 5-year old horse was analysed for *O-*linked glycans using mass spectrometry (Fig. 1B). Monosialylated core-1 *O-*glycans predominated (57.2%), followed by approximately similar concentrations of nonsialylated (22.8%) and disialylated (19%) structures. Core-2 *O-*glycans were detected as <2% of the population of *O-*glycans.

### Lubricin and Galectin Colocalization within the Cartilage Boundary Layer

**Figure 2:**
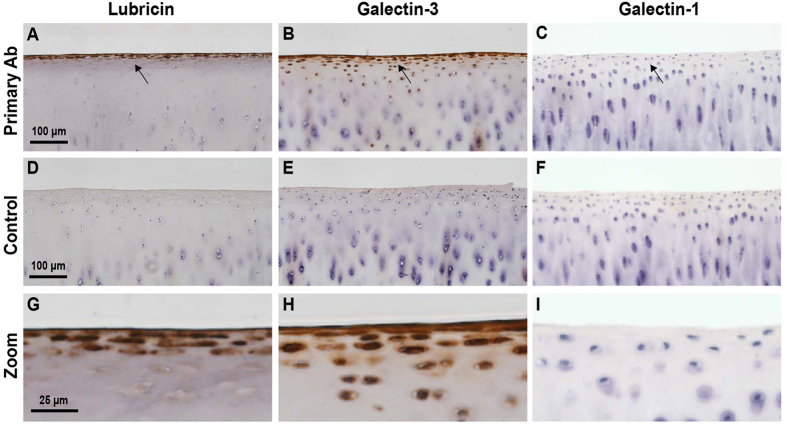
Lubricin and galectin-3 both localize to the cartilage surface. Immunohistochemical detection of lubricin, galectin-3 and galectin-1 in photomicrographs of equine articular cartilage imaged at 20X (**A**–**F**) and magnified 4X (**G**–**I**). (**A**,**G**) Lubricin and (**B**,**H**) galectin-3 are both detected on the boundary layer of articular cartilage, whereas galectin-1 is not (**C**,**I**). Lubricin immunoreaction is observed in superficial zone chondrocytes and as a distinct layer along the lamina splendens (**G**), and galectin-3 immunoreaction is present within both superficial and middle zone chondrocytes and the lamina splendens (**H**). (**D**–**F**) Negative controls. Omission of primary antibody incubation confirms the absence of antigen-independent staining.

Core 2 O-glycans are high affinity ligands for galectin-1^29^, and nonsialylated core 1 O-glycans are high affinity ligands for galectin-3^30^. Based on the observed glycophenotype of lubricin, we predicted that galectin-3 would strongly associate with the cartilage boundary layer. In order to investigate whether synovial fluid galectins may bind to the *O-*linked glycans present within the cartilage lamina splendens, we performed immunohistochemical staining for both lubricin and galectins. Immunostaining of osteochondral sections obtained from healthy equine carpal joints demonstrated intense staining of both lubricin and galectin-3 on the surface of articular cartilage (Fig. 2A,B,G,H), but not galectin-1 (Fig. 2C,I). Control sections incubated without primary antibodies demonstrated the absence of antigen-independent staining (Fig. 2D–F). Lubricin was immunodetected within superficial zone chondrocytes, as has previously been reported in a variety of species^31^,^32^,^33^, with intense staining localized primarily to a three-cell layer thick zone of flattened superficial zone chondrocytes. Lubricin staining was also present in deeper superficial zone chondrocytes, but chondrocytes from the middle and deep zones of cartilage were negative. Galectin-3 was immunodetected in both superficial and middle zone chondrocytes, with sporadic positive cells in deeper layers.

**Figure 3:**
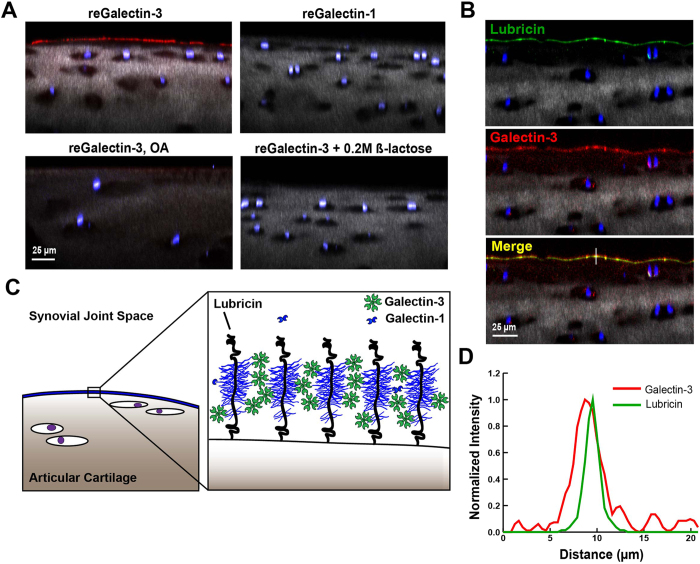
Galectin-3 binds to articular cartilage and colocalizes with lubricin. (**A**) Equine articular cartilage explants imaged using confocal and multiphoton microscopy (40X) following incubation with Alexa647-conjugated galectins. Chondrocyte nuclei are stained with DAPI (blue), and collagen is imaged using second harmonic generation microscopy (grey). Galectin-3 prominently localizes to the boundary layer of healthy cartilage whereas galectin-1 does not. Galectin-3 staining is significantly decreased in joints with severe osteoarthritis (OA) and in the presence of 0.2 M β-lactose, suggesting carbohydrate-specific binding. (**B**) Lubricin stained with anti-lubricin mAb MABT401 and A647-conjugated galectin-3 binding to the surface of articular cartilage, demonstrating colocalization of lubricin and galectin-3. (**C**) Proposed role of galectin-3 in stabilizing the articular cartilage lubricin boundary layer. Pentavalent galectin-3 binds to glycans on adjacent lubricin polymer brushes, providing mechanical stabilization to the boundary layer through lubricin crosslinking. (**D**) Line scan from the white line in B demonstrating colocalization of lubricin and galectin-3 at the level of the articular cartilage boundary layer.

To demonstrate more definitively the colocalization of both lubricin and galectin-3 within the lamina splendens, articular cartilage explants from the femoral condyles of healthy and severe OA equine knee joints were incubated in the presence of Alexa647-conjugated recombinant equine galectins-1 and -3 and/or Alexa568-labeled anti-lubricin antibody. In healthy articular cartilage, lubricin and galectin-3 both intensely stained the boundary layer (Fig. 3A), whereas galectin-1 was nearly undetectable. Carbohydrate-specificity of galectin staining was demonstrated by incubation with a competitive inhibitor, β-lactose, which abolished galectin-3 surface binding. Galectin-3 localization to the lamina splendens was significantly diminished in severely degenerated OA cartilage (Fig. 3A). In severe OA cartilage explants, the second harmonic generation signal within the cartilage matrix was significantly diminished, presumably due to collagen loss. In healthy cartilage explants, lubricin and galectin-3 colocalized to the articular cartilage boundary layer (Fig. 3B), with focal, intense staining confined primarily to the lamina splendens (Fig. 3D). With its ability to form pentamers, galectin-3 has the potential to link multiple lubricin monomers, thereby reinforcing the lubricin-galectin lattice (Fig. 3C).

### Measurement of Lubricin Galectin Binding Kinetics

**Figure 4:**
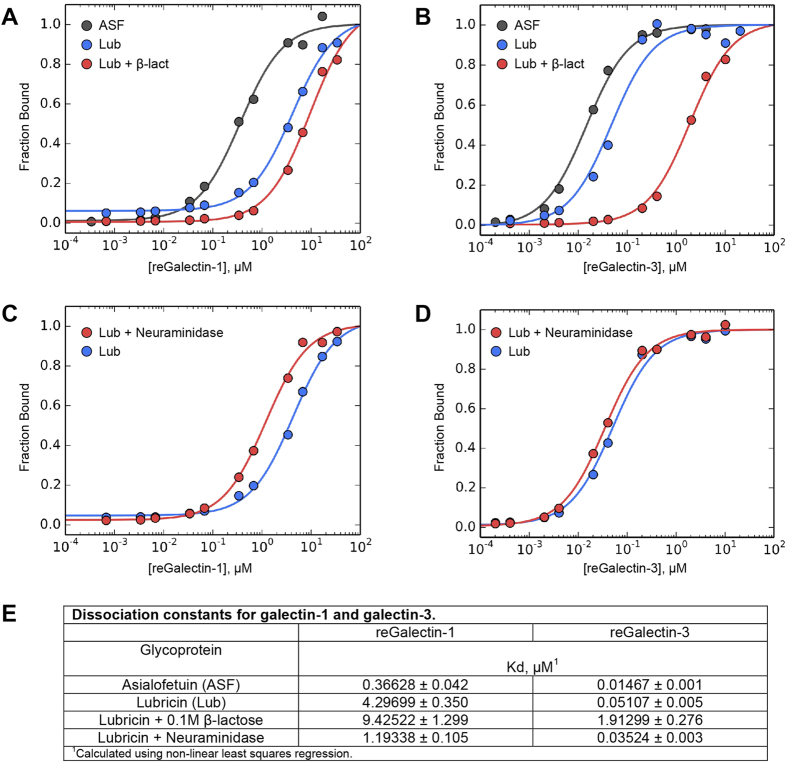
Galectin-3 binds to lubricin with high affinity. Experimental binding curves for (**A**) galectin-1 and (**B**) galectin-3 to asialofetuin (ASF), equine synovial fluid FPLC-purified lubricin, and equine synovial fluid FPLC-purified lubricin +0.1 M β-lactose. Galectin-3 binds to lubricin with high affinity, and this interaction is inhibited by the addition of 0.1 M β-lactose, indicative of carbohydrate-dependent binding. (**C**,**D**) Binding curves for (**C**) galectin-1 and (**D**) galectin-3 to native lubricin vs. sialidase-treated lubricin. (**E**) *K*_*d*_ values for galectin binding to ASF and lubricin. Calculations were performed by non-linear curve fitting with Python. The errors of the fitted Kds are the square root of variances returned by the fitting algorithm.

In order to assess whether synovial fluid galectins may be contributing to the stability and function of the lubricin cartilage boundary layer, we employed a colorimetric binding assay using biotinylated galectins and synovial fluid-purified lubricin. Recombinant equine galectin-3 bound to lubricin with high-affinity, with a measured dissociation constant (*K*_*d*_) of 51 nM (Fig. 4B,E) as compared to 4.3 μM for galectin-1 (Fig. 4A,E). In comparison, asialofetuin, a known high-affinity galectin-binding protein^34^, yielded a 15 nM *K*_*d*_ for galectin-3 binding (Fig. 4B,E) and a 0.36 μM *K*_*d*_ for galectin-1 binding (Fig. 4A,E). Lubricin-galectin binding was carbohydrate-specific, as demonstrated by the approximately 37-fold increase in *K*_*d*_ for galectin-3 in the presence of 0.1 M β-lactose. Removing terminal sialic acid residues from lubricin via incubation in Sialidase A^TM^ (Fig. 4F) increased the affinity of both galectin-1 (Fig. 4C,E) and galectin-3 (Fig. 4D,E) for synovial fluid lubricin, consistent with experimental observations of galectin binding to nonsialylated vs. sialylated variants of biantennary type *N-*glycans^35^. These data suggest that galectin-3, but not galectin-1, can bind to intra-articular lubricin with high affinity and that lubricin-galectin interactions are affected by the glycosylation status of lubricin, specifically terminal sialylation. Given that lubricin sialylation is increased in RA as compared to OA^18^ and that sialylation is increased in OA chondrocytes as compared to healthy chondrocytes^26^, lubricin-galectin interactions may be adversely affected by glycosylation changes that occur in arthritis.

### Galectin-3 Enhances Cartilage Boundary Lubrication

**Figure 5:**
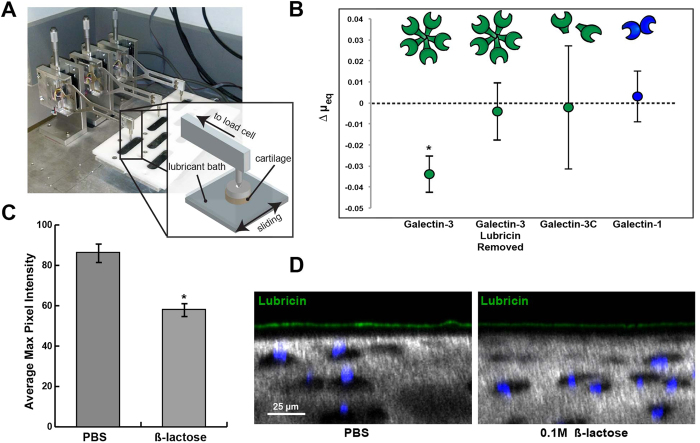
Galectin-3 enhances cartilage boundary lubrication. (**A**) Schematic of custom tribometer used to measure boundary mode frictional coefficients for cartilage on glass in the presence of galectin solutions. (**B**) Galectin-3 decreases equilibrium friction coefficients as compared to paired controls (PBS), but only in the presence of endogenous articular lubricin. When lubricin is extracted using a 30-minute incubation with 1.5 M NaCl, galectin-3 no longer enhances lubrication. The galectin-3C mutant fails to enhance lubrication, suggesting that multimerization is critical for the ability of galectin-3 to facilitate boundary lubrication. Results are presented as mean ± standard deviation (SD) of n = 4. *p < 0.05. (**C**) Average maximum pixel intensity of boundary layer lubricin staining for cartilage explants incubated in either PBS or 0.2 M β-lactose for 12 hrs at 4 °C. Values represent the mean ± standard error (SE) of five independent samples quantified in NIH ImageJ software. Lubricin staining is decreased in the 0.1 M β-lactose treated explants, suggesting a role for galectins in stabilization of the lubricin boundary layer. (**D**) Equine articular cartilage explants incubated in either PBS or 0.2 M β-lactose for 12 hrs at 4 °C, followed by equilibration and incubation with α-lubricin mAb 9G3. Explants are imaged using confocal and multiphoton microscopy (40X). Chondrocyte nuclei are stained with DAPI (blue), and collagen is imaged using second harmonic generation microscopy (grey).

Mechanical functions of galectins in boundary lubrication have not been evaluated. We have previously described a method for measuring equilibrium coefficients of friction (COF) for full-thickness bovine cartilage explants using a cartilage-on-glass custom tribometer^36^. As shown in the schematic (Fig. 5A), the tribometer linearly oscillates a cartilage explant against a polished glass counterface immersed in a PBS or synovial fluid solution. For these experiments, a solution of PBS alone or solutions of PBS with 50 ug/mL of recombinant human galectin-1 and galectin-3 were tested. Galectin-3 decreased equilibrium friction coefficients for cartilage explants by 13% compared to PBS controls (μ = 0.231 ± 0.017 vs. μ = 0.265 ± 0.020, p = 0.0422), but only in the presence of endogenous articular lubricin (Fig. 5B). When surface lubricin was extracted from cartilage explants using a 30-minute incubation in 1.5 M NaCl^33^, followed by re-equilibration in PBS, galectin-3 had no impact on COF (p = 0.83), suggesting that galectin-3 interaction with surface-adsorbed constituents was a prerequisite for cartilage lubrication. Galectin-1 had no effect on cartilage lubrication (p = 0.96).

Galectin-3 has been shown to rapidly precipitate as pentamers in the presence of multivalent ligands^37^ and to crosslink cell surface receptors, resulting in robust galectin-ligand lattices^38^. We next evaluated whether galectin-3 multimerization was necessary to facilitate cartilage lubrication. The galectin-3C truncation mutant, consisting of 143 amino acid residues from the carboxy-terminus of human galectin-3, retains the carbohydrate binding ability of galectin-3 but lacks the amino-terminal domain critical for multivalent behaviour^39^. As opposed to native galectin-3, the galectin-3C multimerization-incompetent mutant had no impact on boundary lubricating function (p = 0.88).

To assess whether multivalent galectin-3 could stabilize surface-adsorbed lubricin by increasing the residence time of adsorbed lubricin, we evaluated articular cartilage lubricin staining after a 12-hour incubation with 100 mM β-lactose. In the presence of β-lactose to compete for galectin binding, lubricin surface staining was diminished by 32.6% (58.2 ± 3.1 vs. 86.4 ± 4.3, n = 5 explants, p < 0.001) (Fig. 5C,D), suggesting that removal of galectins leads to loss of articular lubricin. Taken together, our results suggest that multivalent interactions mediated by galectin-3 stabilize and reinforce the articular cartilage boundary layer, thereby facilitating boundary mode lubrication and protecting against cartilage damage.

## Discussion

The glycosylation of lubricin is essential to its lubricating function. Besides contributing to the overall net negative charge and hydration of the articular cartilage surface layer^9^,^10^,^13^, we demonstrate that lubricin’s carbohydrates are also critical for stabilizing and reinforcing the adsorbed lubricating layer through interactions with galectin-3. Here, we show that galectin-3 is a high-affinity, specific binding partner for lubricin capable of enhancing cartilage boundary lubrication, whereas galectin-1 is not. Furthermore, we demonstrate that boundary lubrication is only enhanced through multimerization-competent galectin-3, supporting the hypothesis that galectin-3 is reinforcing the lubricin boundary layer through formation of a stable lubricin-galectin lattice.

Galectins comprise a family of at least 15 multivalent lectins with specificity for β-galactoside sugars. Prototypical galectins, such as galectin-1, possess a single carbohydrate recognition domain and can form homodimers through N-terminal interactions. Galectin-3 is unique in that it forms pentamers, resulting in a functionally pentavalent carbohydrate-binding molecule. Through glycan binding, galectins can crosslink glycoproteins to form complex lattices on the cell surface or within the extracellular matrix^40^,^41^. Galectins-1 and -3 are expressed in synovial fluid, and altered concentrations of both galectins-1 and -3 have been found in juvenile idiopathic arthritis^42^,^43^ and RA^27^,^44^. Immunohistochemical staining of human OA cartilage has revealed intense staining for galectins-1 and -3 in superficial zone chondrocytes with scarce positivity for galectins-2, -4, -7, -8 and -9^26^. Our studies suggested that equine synovial fluid lubricin is uniquely glycosylated, rich in terminal monosialylated and non-sialylated β-galactoside sugars. Therefore, we hypothesized that the predominant synovial fluid galectins-1 and -3 could be crosslinking lubricin to facilitate lubrication in synovial joints.

Because lubricin and galectin-3 interact in a carbohydrate-dependent manner, glycosylation changes in arthritis may have significant effects on galectin-3 stabilization of the lubricin boundary layer. We are just beginning to understand how lubricin is glycosylated and how that glycosylation is altered in the inflammatory context of arthritis. Early studies revealed that 2/3 of the O-GalNAc-Gal oligosaccharide chains on lubricin were capped with terminal sialic acid residues^45^. Mass spectrometry of human synovial fluid lubricin isolated from RA patients revealed more disialylated than monosialylated species as compared to OA patients, leading the authors to hypothesize that sialylation is upregulated with inflammatory disease severity^18^. Our studies reveal that, in healthy equine synovial fluid, the *O-*linked glycophenotype of lubricin consists of primarily core 1 *O-*linked oligosaccharides that are either mono-, di- or nonsialylated, with monosialylated structures predominating. Glycomic analysis of synovial fluid from horses with normal articular cartilage, structural OA lesions, and osteochondral fragments have also revealed a predominance of monosialylated core 1 *O*-glycans^19^. Galectins and selectins are the two primary endogenous ligands for core 1 *O*-glycans, and both galectins^27^,^44^ and selectins^46^ have been identified in synovial fluid. Our studies specifically evaluated galectin-1 and -3 due to the evidence suggesting that these two galectin family members are directly involved in the pathogenesis of arthritis and may serve as potential therapeutic targets for OA and RA^27^,^42^,^43^,^47^.

The impact of galectins on cartilage lubrication and joint mechanics has never been investigated. Herein, we describe an extracellular role for galectins in joint lubrication and cartilage protection in synovial joints. These findings are important to understand the role of galectins in joint biology. Prior reports have primarily focused on galectins within the synovial environment as mediators of inflammation in RA, juvenile idiopathic arthritis and antigen-induced arthritis in animal models^27^,^42^,^48^,^49^,^50^. Galectin-3 expression is increased in OA chondrocytes^51^, and increased galectin-3 immunohistochemical staining has been demonstrated at sites of synovial and cartilage invasion by immune cells in RA and during inflammatory phases of OA^27^, leading authors to hypothesize that galectin-3 is both involved in inflammation and a novel marker of disease activity in RA. Recent lectin staining of human OA cartilage has revealed galectin-3 localization to OA chondrons and interterritorial matrix in severely degenerated cartilage^52^. However, the exact role of galectins and how they contribute to the pathogenesis of disease is not well understood.

Galectins function distinctly through intracellular and extracellular pathways, with intracellular signalling resulting from N-terminal protein-protein interactions and extracellular functions arising primarily from carbohydrate-dependent interactions on the cell surface or within extracellular matrices^53^. One report indicates that intracellular localization of galectin-3 protects chondrocytes from apoptosis and that the absence of galectin-3 in knockout mice leads to the development of OA-like cartilage lesions^54^. Our results describe a biomechanical role for galectin-3 in stabilizing the lubricin boundary layer, which suggests that galectin-3 may promote normal cartilage lubrication and joint homeostasis. It is possible that galectins have dual functions and/or concentration-dependent bimodal effects in synovial joints, with the potential to promote the development of arthritis through immune cell invasion and inflammation^27^,^48^,^55^, but also to protect against the development of cartilage damage and ensuing arthritis by enhancing cartilage lubrication.

Our studies revealed that galectin-3 localized to the boundary layer of healthy articular cartilage and within superficial and middle zone chondrocytes. Although galectin-3 staining has been previously demonstrated in synovial tissues^27^ and chondrocytes from human OA patients^51^,^52^, galectin-3 has not been previously documented to stain the surface layer of articular cartilage. Differences in antibody reactivity or tissue preparation may explain observed differences in galectin boundary layer immunohistochemical staining, with bulky molecules like hyaluronic acid potentially interfering with epitope exposure in the absence of hyaluronidase treatment. The surface layer is resistant to complete digestion by hyaluronidase^56^, and hyaluronic acid is a bulky glycosaminoglycan known to interact with lubricin^57^. Also, galectin-3 may not stain the boundary layer of mildly or severely degenerated OA cartilage due to disruption of the lamina splendens, including the surface-adsorbed lubricin layer, as is observed with lubricin immunolocalization in OA cartilage^22^,^32^. In fact, our imaging results suggest that galectin-3 staining of the articular cartilage surface in severe OA cartilage explants is almost completely abolished. These findings, coupled with the high binding affinities for lubricin and galectin-3 and the colocalization of fluorophore-labelled galectin-3 and lubricin mAb on the lamina splendens suggest that galectins are binding predominantly to lubricin. Although our results do not exclude the possibility that galectin-3 may be binding to glycans on other cartilage surface constituents, such as fibronectin, the sparse distribution of *N-*glycans on the cartilage surface as demonstrated by lectin staining suggests that *O-*linked glycans are the primary targets for galectin binding. Binding of galectin-3 to the core 1 *O-*glycan structure has been characterized previously, with the CRD demonstrating a unique Glu-water-Arg-water motif-based mode^58^. The affinity for Gal-3 was shown to be two orders of magnitude higher than for Gal-1 due to a Galectin-1 pentad residue motif (^5′^AHGDA^55^) that produced significant steric hindrance to core 1 *O-*glycan binding^58^.

Herein, we provide a mechanism to explain how synovial fluid galectin-3 may enhance the biomechanics of cartilage lubrication and protect against the development of cartilage damage through stabilization of the lubricin boundary layer via lattice formation. Future investigation into how lubricin glycosylation is altered in arthritis and how these alterations impact galectin-3 binding and cartilage lubrication will be critical in fully understanding the role of galectins in cartilage biomechanics.

## Materials and Methods

### Ethics Statement

All experimental protocols were approved by the Cornell University Institutional Animal Care and Use Committee (Protocol Number: 2011-027), and all methods were carried out in accordance with approved guidelines.

### Synovial fluid purification and glycophenotyping

Synovial fluid was obtained from healthy joints of two young horses and purified using diethylaminoethyl (DEAE) affinity chromatography using previously described methodology^59^, with some modifications. The O-linked glycans from equine synovial fluid lubricin from healthy carpal joints were characterized with mass spectrometry. Lubricin and galectin immunohistochemistry was performed on osteochondral sections from healthy equine middle carpal joints, and confocal and multi-photon imaging of equine femoral condyle cartilage explants was performed to assess fluorescent lectin binding and lubricin and galectin binding.

### Galectin cloning and recombinant production

RNA was purified from equine kidney tissue, and gene-specific primers were designed against the NCBI predicted sequences of equine galectin-1 and galectin-3. Recombinant human galectin-1 and galectin-3 constructs were obtained from C. Bertozzi and, along with the galectin-3C mutant and equine galectins, were recombinantly expressed and purified using β-lactosyl sepharose affinity chromatography similar to previously described methods^60^.

### Lubricin galectin binding kinetics

Lubricin purified from normal equine synovial fluid or bovine asialofetuin was coated to high-binding, 96-well ELISA plates. Recombinant, biotinylated equine galectin-1 or galectin-3 was added to each well at serial concentrations, and affinities were measured using streptavidin-HRP, TMB and a monochromator.

### Lubricin deglycosylation

FPLC-purified equine synovial fluid lubricin was digested with the following combinations of deglycosylation enzymes: i) N-glycanase^®^ PNGase F, ii) Sialidase A^TM^, or iii) O-glycanase^®^ (Endo-α-N-Acetylgalactosaminidase) + prO-LINK Extender^TM^ (β(1–4) Galactosidase + β-N-Acetylglucosaminidase) (PROzyme^®^, Glyko^®^, Hayward, CA) listed in S1 Table.

### Cartilage tribometry

Articular cartilage explants were aseptically harvested from the femoropatellar groove of young bovine stifles and incubated in PBS or recombinant human galectin-1, galectin-3, or galectin-3C. Tribological testing was performed using a previously described custom friction apparatus^36^.

## Additional Information

**How to cite this article**: Reesink, H. L. *et al*. Galectin-3 Binds to Lubricin and Reinforces the Lubricating Boundary Layer of Articular Cartilage. *Sci. Rep.* **6**, 25463; doi: 10.1038/srep25463 (2016).

## Supplementary Material

Supplementary Information
