# Supplementary material for: Galectin-3 Binds to Lubricin and Reinforces the Lubricating Boundary Layer of Articular Cartilage
Source: Sci Rep. 2016 May 9;6:25463. doi: 10.1038/srep25463 (PMC4860590; doi:10.1038/srep25463)
Supplement: Supplementary Information [file srep25463-s1.pdf]

Galectin-3 Binds to Lubricin and Reinforces the Lubricating Boundary Layer of Articular Cartilage

Heidi L. Reesink[1*], Edward D. Bonnevie[2], Sherry Liu[1], Carolyn R. Shurer[3], Michael J. Hollander[3], Lawrence J. Bonassar[2, 4], Alan J. Nixon[1*]

Supporting Information

Materials and Methods

Ethics Statement

Tissue harvesting protocols for this study were approved by the Cornell University Institutional Animal Care and Use Committee (Protocol Number: 2011-027). All sample collection was performed following humane euthanatization of horses using sodium pentobarbital. For mechanical testing, cartilage from calf knees was obtained from a local abattoir. The proper euthanasia methodology for the abattoir was approved by the USDA.

Purification of Equine Synovial Fluid Lubricin

Following euthanasia via overdose of sodium pentobarbital, the elbow, carpal, fetlock, tarsal and stifle joints of two young (4 and 6-year-old) horses free from clinical signs of joint disease were clipped and aseptically prepared with povidone iodine scrub (Betadine, Purdue Pharma L.P., Stamford, CT) and alcohol. Synovial fluid was aspirated from each joint using an 18 gauge, 3.5-inch spinal needle (BD Medical, Franklin Lakes, NJ) and placed in 15mL

polypropylene conical tubes for clarification via high-speed centrifugation at 10,000xg for 1 h. The synovial fluid supernatants were transferred to fresh polypropylene tubes and stored at -80°C. Clarified synovial fluid was thawed, pooled and purified using previously described methodology,[1] with some modifications. Equine synovial fluid was concentrated five-fold using 100kD molecular weight cut off centrifugal filter devices (Amicon® Ultra, Millipore, Tullagreen, Carrigtwohill, IRL) by centrifugation at 4,000xg, 4°C. The mucinous synovial fluid retentates were re-suspended in 50mM NaAc buffer, pH 5.5, with proteolytic inhibitors, to the original synovial fluid volume. Hyaluronic acid digestion was performed using Streptomyces hyaluronidase (Calbiochem®, EMD Chemicals, San Diego, CA) at a final concentration of 1U/mL of re-suspended synovial fluid at 4°C for 16 h on an end-over-end rocker. The hyaluronidase-digested synovial fluid samples were centrifuged at 4,000xg, 4°C for 5 minutes to pellet debris, and the supernatant was purified using a HiTrap™ diethylaminoethyl (DEAE) fast flow sepharose column (GE Healthcare Life Sciences, Little Chalfont, UK) for fast protein liquid chromatography (FPLC). Lubricin was eluted from the DEAE column after rinsing with 50mM NaAc using a 200mM step-wise elution gradient from 200mM to 1M NaCl. Each 200mM fraction was loaded onto a 4-20% tris-glycine gradient gel (Bio-Rad, Hercules, CA) for reducing and non-reducing sodium dodecyl sulphate polyacrylamide gel electrophoresis (SDS-PAGE), followed by Coomassie staining (Teknova, Hollister, CA). The 400-600mM fraction with a single prominent band at >250kD was dialyzed against phosphate buffered saline (PBS) with proteolytic inhibitors

at 4°C for 36 h, with 3 exchanges of PBS. Immunoblotting of all FPLC-purified fractions was performed using a polyclonal antibody raised in rabbit and directed against the synthetic peptide CLPNIRKQPDGYDYYAFSKDQ corresponding to amino acids 1356-1374 of the C-terminus of human lubricin (ab28484, Lot: GR116636-3, Abcam®, Cambridge, UK), in addition to the monoclonal antibody 9G3 raised in mouse and directed against the mucin domain of human lubricin (MABT401; EMD Millipore), which is highly conserved across species. Both antibodies were predicted to cross-react with equine lubricin based on sequence homology of the C-terminus and mucin-rich region of lubricin, and antibodies were validated in equine using synovial fluid as a positive control and serum as a negative control. The final protein concentration of lubricin fractions was quantitated using a bicinchoninic acid protein assay (Thermo Scientific, Rockford, IL) using bovine serum albumin as the standard and read out on a microplate reader (Tecan Safire, Mannedorf, Switzerland) at 562nm absorbance.

MALDI/TOF-MS Glycosylation Analysis of Synovial Fluid Lubricin

For lubricin glycosylation analysis, synovial fluid was aspirated in similar fashion from the carpal joints of a healthy 5-year old horse and clarified by centrifugation. The >100kD synovial fluid retentate was digested at with 1U/mL of Streptomyces hyaluronidase in 50mM NaAc buffer, pH 5.5 with proteolytic inhibitors overnight at 4°C. Lubricin was purified using DEAE anion-exchange resin, rinsing with 350mM NaCl and eluting in 1M NaCl. The purified lubricin was de-salted

overnight using a 50kD tube dialyzer (G-Biosciences, St. Louis, MO), followed by removal of fibronectin via gelatin separopore® 4B purification (Bioworld, Dublin, OH) and elution in ultrapure water. For mass spectrometry, the lubricin samples were dried and subjected to beta-elimination to cleave O-linked glycans. O-linked glycans were cleaved with NaOH and serine-linked GalNAc was reduced with $NaBH_4$. The reaction was neutralized with acetic acid, desalted, and cleaned of borates prior to permethylation. O-linked glycans were permethylated for structural characterization by mass spectrometry, dissolved with methanol and crystallized with α-dihydroxybenzoic acid, 20mg/mL, in 50% methanol: water. Glycan analysis was performed in the positive ion mode by MALDI-TOF/TOF-MS using AB SCIEX TOF/TOF 5800 (Applied Biosystems, Thermo-Scientific, Waltham, MA).

Lubricin and Galectin Cartilage Immunohistochemistry

Osteochondral blocks were harvested from the carpal bones of the middle carpal joint of a healthy 4-year old horse, fixed in 4% paraformaldehyde, de-calcified in 10% EDTA for three weeks and embedded in paraffin. Thin (5 μm) sagittal sections were obtained for immunohistochemical staining of lubricin and galectins. Sections were deparaffinized, re-hydrated in three changes of xylene and serial alcohol, followed by treatment with 1% hyaluronidase (Sigma-Aldrich, St. Louis, MO) solution in 20mM sodium acetate at 37°C for 30 min. Endogenous peroxidase activity was quenched by treatment with 3% hydrogen peroxide for 30 min. After blocking in normal goat or rabbit serum, a mouse

monoclonal antibody against lubricin (MABT401; EMD Millipore) or a goat polyclonal antibody against galectin-3 (sc-19280; Santa Cruz) was added at 1:200 or 1:100 dilutions and incubated at room temperature for 1 hr. After three washes in PBS-T (0.1%), the sections were incubated with a biotinylated goat anti-mouse IgG (Vectastain, Vector Labs) or a biotinylated rabbit anti-goat IgG (Vectastain, Vector Labs). After 3 rinses in PBS, immunodetection was performed using the Vectastain ABC Kit and ImmPACT DAB reagent (Vector Laboratories). The sections were rinsed in PBS, counterstained using haematoxylin (Fisher) and imaged with a 20x objective using an Olympus DP80 (Olympus) camera and an Olympus IX73 microscope (Olympus).

Confocal and Multi-photon Imaging of Cartilage Explants

Articular cartilage biopsies were harvested from the medial femoral condyle of adult equine stifle joints using a 6mm biopsy punch (Miltex, York, PA) and immediately frozen at -80°C. For lectin imaging, cartilage explants were thawed to room temperature, rinsed in PBS and incubated for 1 hour at 4°C in 10ug/mL FITC-conjugated lectin (Vector Labs, Burlingame, CA), or Atto488-conjugated MAL-II lectin. MAL-II (Vector Labs) was conjugated with Atto488 in the presence of 150mM β-lactose. Explants were rinsed in PBS and stained with 1ug/mL Hoechst 33342 (Life, Thermo Scientific, Waltham, MA) for 20 minutes to stain chondrocyte nuclei, followed by several rinses in PBS. Explants were imaged in glass dishes (MatTek, Ashland, MA) using a Zeiss inverted laser scanning microscope with a 40X water immersion objective. For fluorescent galectin

and/or lubricin staining, explants were first incubated for 1 hour at 4°C in 100mM β-lactose to extract endogenous galectins. Following re-equilibration in PBS, each explant was incubated in 10ug/mL of A647-labeled galectin-1 or galectin-3 in PBS for 1 hour at room temperature. As a control for non-specific binding, explants were also incubated with A647-labeled galectin-1 or galectin-3 in the presence of 300mM β-lactose. For lubricin staining, explants were incubated in 10ug/mL of A568-labeled anti-lubricin mAb (MABT401, EMD Millipore). To compare lubricin staining following β-lactose removal of galectins, explants were incubated in either PBS or PBS + 100mM β-lactose at 4°C for 12 hours, followed by 3 rinses in PBS, incubation in 10ug/mL of A568-labeled anti-lubricin mAb for 1 hour, and Hoechst staining. Explants were rinsed in PBS, and z-stacks were obtained for four regions of interest on five separate explants for quantitation. All experiments were performed in triplicate.

Cloning, Expression and Purification of Galectins

Human galectin-1, galectin-3 and galectin-3C

The N-terminal truncation mutant, Gal-3C, was made through PCR amplification of a human Gal3 template with forward (5’-GGCAGGATCCTACCCTGGAGCACCTGGAGCTTATC -3’) and reverse (5’-GGCAGCGGCCGCTTATATCATGGTATATGAAGCACTG-3’) primers that introduced 5’ BamHI and 3’ NotI restriction sites for cloning into pET21a. Recombinant human galectin-1 and galectin-3 constructs were obtained from C. Bertozzi and, along with the galectin-3C mutant, were expressed in XL1-Blue

competent *E. coli*. Recombinant galectins were purified using β-lactosyl sepharose affinity chromatography similar to previously described methods[2]. Briefly, Sepharose® 6B (Sigma-Aldrich, St. Louis, MO) was activated with 40% divinyl sulfone prepared in 0.5M Na_2CO_3, pH 11.0, followed by washing with distilled, deionized water and 0.5 M Na_2CO_3, pH 10.0, and conjugation with 40% β-lactose (Santa Cruz Biotechnologies, Santa Cruz, CA) in 0.5 M Na_2CO_3, pH 10.0. The conjugated lactosyl sepharose was incubated with 0.5M Na_2CO_3, pH 8.8 plus β-mercaptoethanol and equilibrated in PBS, pH 7.5 for storage at 4°C prior to use. Bacterial lysates were incubated with 20mL of β-lactosyl sepharose slurry per litre volume of bacterial culture in a 50mL conical tube for 3 h at 4°C. The slurry-lysate mixture was loaded onto a 100mL gravity column, and washed 3 times with 3 column volumes of PBS + 8mM dithiothreitol (DTT). Galectins were eluted from the lactosyl sepharose resin with 1.5 column volumes of 0.1M β-lactose + 8mM DTT, followed by concentration with 3K (galectin-1) or 15K (galectin-3 and -3C) molecular weight cut off centrifugal filter devices (Amicon® Ultra, Millipore, Tullagreen, Carrigtwohill, IRL) to 3mL final volume. Galectins were centrifuged at 13,000rpm for 10 minutes, and the supernatant was loaded onto a HiLoad® 16/60 Superdex® 200 gel filtration column (GE Healthcare Life Sciences, Little Chalfont, UK) for FPLC. Fractions were analysed by SDS-PAGE, followed by Coomassie staining (Teknova, Hollister, CA), and pure fractions yielding a 15kD band for galectin-1 or a 31kD band for galectin-3 were pooled and concentrated to 8mg/mL based on 280nm absorbance (0.602 for galectin-1 and 1.372 for galectin-3) for storage in 0.1M β-lactose + 8mM DTT at -

80°C.

Equine galectin-1 and galectin-3

cDNA was isolated from kidney tissue harvested from a 19-year-old Thoroughbred mare following euthanatization. RNA was purified following renal tissue homogenization and lysis with Proteinase K (PerfectPure, 5PRIME, Gaithersburg, MD), followed by cDNA synthesis (SuperScript® First-Strand Synthesis, Invitrogen, Carlsbad, CA).

Gene-specific primers were designed against the predicted sequences of equine galectin-1 and galectin-3 from the National Center for Biotechnology Information (NCBI), with the equine galectin-1 forward primer designed to begin at nucleotide (nt) 292 of the predicted equine sequence based upon sequence homology between the human homologue, translated as MACGLVASNLNKPGECL and the fact that the predicted equine galectin-1 mRNA sequence was 292bp longer than the human homologue. Thus, the galectin primers were designed as follows:

(Galectin-1 Fwd: 5'- ATGGCTTGTGGTCTGGTCGCCAGCA –'3,

Galectin-1 Rev: 5'- TCACTCAAAGGCCACACACTTGATCT –3',

Galectin-3 Fwd: 5'- ATGTCAGACGGTTTTTCGCTTA –3',

Galectin-3 Rev: 5'- TTATATCATAGTGTGCGAAGCACTG –3').

Bands corresponding to 408bp for galectin-1 and 718bp for galectin-3 were gel purified (QIAquick, QIAGEN GmbH, Hilden, Germany) and sub cloned from the

pCR^{TM}2.1-TOPO® vector into a pET21a bacterial expression vector, followed by sequencing by the Cornell University Biotechnology Resource Center. Following sequencing, equine galectins-1 and -3 were recombinantly expressed in XL1-Blue competent *E. coli* and purified using lactosyl sepharose chromatography and FPLC gel filtration as previously described for the purification of human galectins-1, -3 and -3C. Equine galectins were concentrated to 8mg/mL based on 280nm absorbance, using extinction coefficients of 0.600 for galectin-1 and 1.336 for galectin-3 predicted from the equine galectin sequences on Expasy's ProtParam online tool. Following concentration, equine galectins were stored in 0.1M β-lactose + 8mM DTT and snap frozen at -80°C.

Measurement of Lubricin Galectin Binding Kinetics

Biotinylation of equine galectins

Recombinant equine galectin-1 and -3 were biotinylated in the presence of 200mM β-lactose + 8mM DTT to preserve the carbohydrate recognition domain binding capacity of galectins using an EZ-LINK^{TM} NHS-PEG$_4$-Biotinylation Kit according to manufacturer instructions (Thermo Scientific, Rockford, IL). Following desalting, a small aliquot of galectins were saved in PBS for quantification of biotin incorporation by measuring the change in 500 nm absorbance before and after the addition of biotinylated galectins to HABA/Avidin Solution. The remaining biotinylated galectins were re-suspended in 200mM β-lactose + 8mM DTT and snap frozen at -80°C prior to performing binding assays. A HABA microplate assay was used to quantify equine galectin biotin

incorporation, measured as 1.42 biotin molecules per molecule of galectin-1 and 2.63 biotin molecules per molecule of galectin-3.

Colorimetric plate assay

Lubricin purified from normal equine synovial fluid or bovine asialofetuin (Sigma-Aldrich, St. Louis, MO) was coated to high-binding, 96-well ELISA plates (Corning Inc., Corning, NY) in sodium carbonate buffer, pH 9.6, at 4°C and at a final concentration of 4ug/mL. After incubation for 16 hours, wells were aspirated and rinsed in PBS plus 0.1% Tween, followed by blocking with Pierce® Protein-Free (PBS) Blocking Buffer (Thermo Scientific, Rockford, IL). Recombinant, biotinylated equine galectin-1 or galectin-3 was added to each well in duplicate at concentrations ranging from 0.078ug/mL to 5ug/mL in PBS-T (0.1%) for 1hr at room temperature, with and without β-lactose (Santa Cruz Biotechnologies, Santa Cruz, CA) at concentrations of 50, 150 or 500mM. After aspiration and rinsing in PBS-T (0.1%), high-sensitivity streptavidin-HRP (Thermo Scientific, Rockford, IL) was added at a 1:10,000 dilution in PBS-T (0.1%) for 30 minutes, followed by a final aspiration and rinse in PBS-T (0.1%). Finally, trimethylbenzidine reagent (Pierce, Rockford, IL) was added for 10 minutes, the reaction was stopped with 2N H_2SO_4, and absorbance was measured at 450nm with 540nm subtraction. Binding assays were performed similarly for FPLC-purified equine synovial fluid lubricin digested with Sialidase A™ (Glyko).

Lubricin Deglycosylation

Enzymatic reactions

Buffer exchange of FPLC-purified equine synovial fluid lubricin into ultrapure water was performed using spin desalting columns (Zeba™, Thermo Scientific, Rockford, IL), followed by concentration to 1mg/mL with 100K molecular weight cut off centrifugal filter devices (Amicon® Ultra, Millipore, Tullagreen, Carrigtwohill, IRL). Concentrated, buffer-exchanged purified lubricin was digested with the following combinations of deglycosylation enzymes: i) N-glycanase® PNGase F, ii) Sialidase A™, or iii) O-glycanase® (Endo-α-N-Acetylgalactosaminidase) + prO-LINK Extender™ (β(1-4) Galactosidase + β-N-Acetylglucosaminidase) (PROzyme®, Glyko®, Hayward, CA) listed in **S1 Table**. Deglycosylation reactions were carried out according to the non-denaturing protocol in the manufacturer's instructions, using 50μg of lubricin per reaction in the presence of 0.25M sodium phosphate, pH 7.0 at 37°C for 16 hrs.

Electrophoresis and SYPRO staining

Following deglycosylation, 5ug (10uL) of native lubricin and each lubricin reaction were resolved on a NuPAGE® Novex® 3-8% Tris-Acetate gel under denaturing conditions. Bands were detected using fluorescent SYPRO® Ruby gel stain and compared to HiMark™ pre-stained high molecular weight standards (Invitrogen, Carlsbad, CA) to assess the extent of deglycosylation.

Cartilage Tribometry

Articular cartilage explants were aseptically harvested from the femoropatellar

groove of young bovine stifles and stored in PBS at -20°C prior to tribological testing. After thawing to room temperature, cylindrical samples (6 mm diameter by 2 mm high) were incubated in sterile PBS or recombinant human galectin-1, galectin-3, or galectin-3C at a concentration of 50μg/mL for 1 h. Cartilage samples were submerged in a bath of PBS or galectin for tribological testing using a custom friction apparatus. This testing method has been previously described[3]. Briefly, the friction apparatus linearly oscillated each sample against a glass counterface three times at a controlled speed of 0.32mm/sec after allowing the cartilage sample to relax for 1 hour under an applied normal strain of 30%. Custom Matlab code (The Mathworks, Natick, MA) was used to calculate the mean equilibrium frictional coefficient (μ_{eq}) based on the final two oscillations. Tribometry data were compared using 2-sample student's t-tests with $p < 0.05$ considered significant, and all values are reported as mean ± SEM.

Results

Purification of Synovial Fluid Lubricin

Coomassie staining of equine diethlyaminoethyl (DEAE) FPLC-purified synovial fluid fractions subjected to SDS-PAGE on a 4-20% tris-glycine gel revealed a single, prominent protein band at >250kD for the 400-600mM fraction, consistent with equine lubricin **(S1 Fig A)**. Faint bands were present at ~70kD and 25kD, and immunoblotting with a C-terminal lubricin antibody (ab28484, Abcam) showed reactivity with the >250kD, 70kD and 25kD bands **(S1 Fig B)**. Similar

70kD bands, consistent with a minimally glycosylated PRG4 fraction containing both N- and C-terminal fragments, have been detected following 1.0 NaCl DEAE affinity purification of bovine synovial fluid upon silver staining and immunoblotting[4]. Lower molecular weight bands in the 37-75 kD range have been detected in the synovial fluid of human OA and RA patients[5], suggesting that lubricin is subject to cleavage. SyPRO Ruby® staining of DEAE FPLC-purified equine synovial fluid on a 3-8% tris-acetate gel performed under denaturing conditions yielded two major >460kD bands, including a higher molecular weight smear, consistent with a species with variable glycosylation **(S2 Fig).** Heavily glycosylated proteins are known to run as smears during gel electrophoresis due to the heterogeneity of glycosylation and imperfect charge-to-mass ratio that occurs with bulky, charged terminal sugars[6]. Sialidase treatment results in a slight reduction in molecular size of lubricin, with a more dramatic reduction in the presence of both O-glycosidase and sialidase.

Supporting Information Captions

S1 Fig. Purification of equine synovial fluid lubricin. (A) FPLC chromatogram and (**B)** Coomassie and immunoblot analyses of equine lubricin in FPLC DEAE anion exchange fractions. SDS-PAGE was performed using 4-20% tris-glycine gradient gels. Immunoblotting was performed with an anti-lubricin antibody (ab28484; Abcam) under non-reducing conditions.

S2 Fig. Deglycosylation of equine synovial fluid lubricin. SYPRO Ruby®-stained 3-8% tris-acetate gel with FPLC-purified equine lubricin and lubricin treated with sialidase, N-glycanase and O-glycosidase + sialidase.

S1 Table. Enzymes used to deglycosylate equine synovial fluid lubricin.

**Table 1. Deglycosylation Enzyme Functions**	
**Enzyme**	**Function**
N-Glycanase® (PNGase F)	Removes all N-glycans
Sialidase A™	Removes sialic acids
O-Glycanase®	Removes O-glycans after removal of extended structures
β(1-4)-Galactosidase	Removes glycans (e.g. polylactosamines)
β-N-Acetylglucosaminidase	Removes glycans (e.g. polylactosamines)

References

1. Teeple, E. *et al.* The Effects of Supplemental Intra-Articular Lubricin and Hyaluronic Acid on the Progression of Post-Traumatic Arthritis in the Anterior Cruciate Ligament Deficient Rat Knee. *Am. J. Sports Med.* **39,** 164–172 (2011).

2. Pace, K. E., Hahn, H. P. & Baum, L. G. Preparation of recombinant human galectin-1 and use in T-cell death assays. *Methods Enzymol.* **363,** 499–518 (2003).

3. Gleghorn, J. P., Jones, a R. C., Flannery, C. R. & Bonassar, L. J. Boundary mode frictional properties of engineered cartilaginous tissues. *Eur. Cell. Mater.* **14,** 20–8; discussion 28–9 (2007).

4. Schmidt, T. a, Plaas, A. H. K. & Sandy, J. D. Disulfide-bonded multimers of proteoglycan 4 PRG4 are present in normal synovial fluids. *Biochim. Biophys. Acta* **1790,** 375–84 (2009).

5. Estrella, R. P., Whitelock, J. M., Packer, N. H. & Karlsson, N. G. The glycosylation of human synovial lubricin: implications for its role in inflammation. *Biochem. J.* **429,** 359–67 (2010).

6. El-Rassi, Z. *Carbohydrate Analysis by Modern Chromatography and Electrophoresis*. (Elsevier, 2002). at <https://books.google.com/books?id=xrHDySs1NZUC&pgis=1>

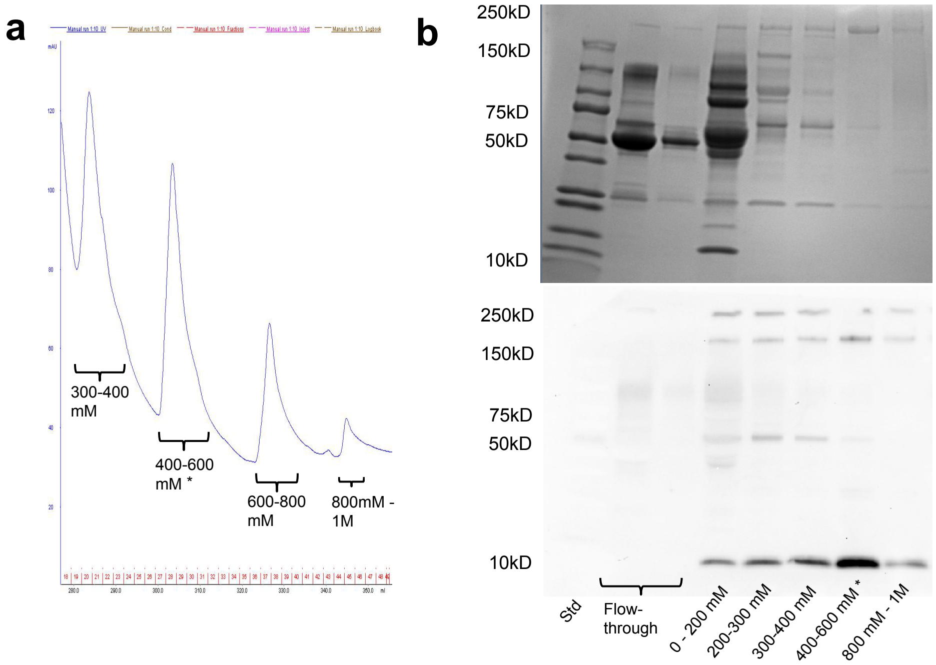
a
300-400 mM
400-600 mM *
600-800 mM
800mM - 1M
b
250kD
150kD
75kD
50kD
10kD
250kD
150kD
75kD
50kD
10kD
Std
Flow-through
0 - 200 mM
200-300 mM
300-400 mM
400-600 mM *
800 mM - 1M

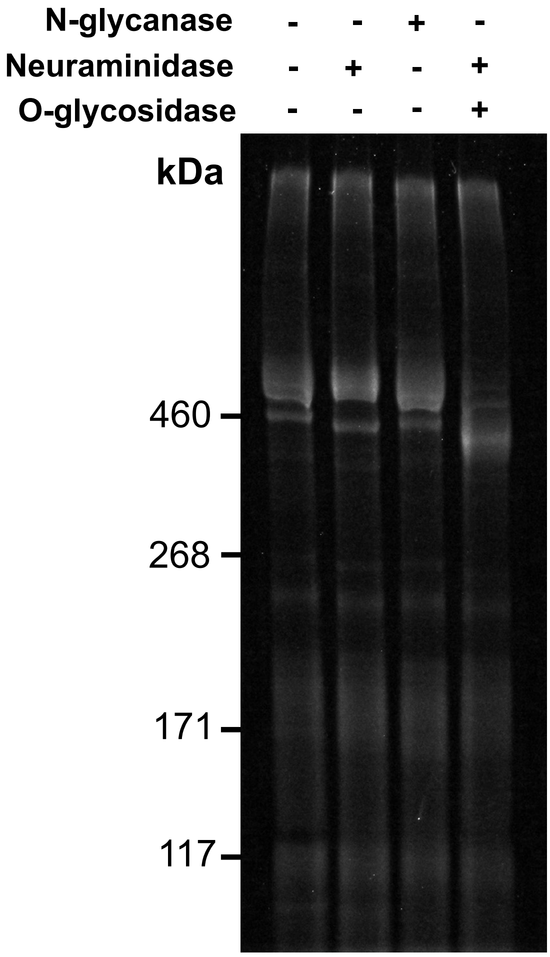
N-glycanase - - + -
Neuraminidase - + - +
O-glycosidase - - - +
kDa
460
268
171
117
